# ESPO and Behavioral and Social Sciences Section Symposium: Addressing the Needs of Black, Indigenous, and People of Color (BIPOC) Communities Throughout the Stage Model

**DOI:** 10.1093/geroni/igab046.1232

**Published:** 2021-12-17

**Authors:** Briana Sprague, Kylie Meyer, Chivon Mingo

## Abstract

Behavioral interventions have been successfully deployed to prevent and manage chronic conditions among older persons, improve mental health, and support caregivers’ ability to cope with care stressors. However, intervention effects may not be equally distributed among populations, nor equally acceptable or accessible among Black, Indigenous, and People of Color (BIPOC) communities. In this symposium, we will discuss how behavioral interventions can better meet the needs of BIPOC older adults and caregivers, who may not equally benefit from advancements in behavioral interventions due to issues such as a reliance on non-diverse study samples and lack of cultural tailoring. This symposium will be structured in accordance with the National Institutes of Health Stage Model of Behavioral, and will feature researchers whose work address BIPOC needs across the trajectory of intervention development. Representing Stage 1 research, Fayron Epps, PhD, RN, will describe her use of a community advisory council to develop a faith-based toolkit to support African Americans living with dementia and their caregivers. Next, Laura Gitlin, PhD, MA, will describe her experiences testing a Stage 3 intervention to lower depression among African Americans, including challenges advancing the culturally-tailored program to Stage 4. Lastly, Shanae Rhodes, BSN, RN will describe her Stage 2 evaluation of a conversation group created and attended by women of color to socially connect in response to COVID-19. Although speakers will describe research projects that represent specific research Stages, this symposium will have a large discussion-based component and will cover all parts of the Stage Model of Behavioral Intervention.

